# Co‐Creating Supportive Leadership and Nurse Work Environments: A Mixed‐Methods Participatory Action Research Study in Saudi Arabia

**DOI:** 10.1155/jonm/7257178

**Published:** 2026-09-27

**Authors:** Majed Mowanes Alruwaili

**Affiliations:** ^1^ Department of Nursing Administration and Education, College of Nursing, Jouf University, Sakaka 72388, Saudi Arabia, ju.edu.sa

**Keywords:** burnout, leadership, nurses, professional, Saudi Arabia, workplace

## Abstract

**Background:**

Co‐created leadership may improve nurses’ work environments, but evidence on candidate pathways in hierarchical settings is limited.

**Aim:**

To evaluate processes, outcomes and exploratory change pathways associated with a participatory supportive leadership programme in Saudi hospitals.

**Methods:**

We conducted a five‐hospital participatory action research evaluation with an uncontrolled three‐wave cohort (*N* = 200), mixed‐effects outcome models, exploratory bootstrap product‐of‐coefficients analyses, nine interaction tests with Benjamini–Hochberg false‐discovery‐rate control and longitudinal qualitative data.

**Results:**

Retention was 86.0%. Practice Environment Scale of the Nursing Work Index scores improved (*d* = 0.36), while personal and work‐related burnout and turnover intention decreased and job satisfaction increased; client‐related burnout did not change significantly. Thirteen of 20 unadjusted change‐pathway intervals excluded zero. Four interactions were nominally significant, but none survived false‐discovery‐rate correction. Complete‐case analyses preserved the significance classification for 10 of 11 outcomes; staffing/resource adequacy attenuated (*p* = 0.038 to 0.054). Qualitative themes contextualised, but did not confirm, quantitative patterns.

**Conclusions:**

The programme was associated with favourable relational and participatory changes, but the uncontrolled design precludes causal or mediation claims.

**Implications for Nursing Management:**

Managers can use structured co‐creation to support nurse voice and psychological safety while pairing local leadership action with staffing policy and readiness‐sensitive implementation.

**Accessible Summary**
• What is already known about this topic:
◦ Supportive leadership is a modifiable determinant of nurses’ work environments, burnout and turnover intention.
◦ Participatory action research (PAR) can structure leadership change with frontline nurses, but candidate pathways and contextual influences remain uncertain.
◦ Evidence from hierarchical, resource‐constrained healthcare settings remains limited.
• What this paper adds:
◦ Psychological safety and perceived voice had the largest number of exploratory concurrent change‐pathway intervals excluding zero; these analyses do not establish mediation.
◦ Four of nine contextual interaction tests met nominal *p* < 0.05 (staffing burden, hierarchy intensity and two organisational readiness terms); none survived false‐discovery‐rate correction, and their directions require confirmation.
◦ Nurses described gains in voice and ownership alongside persistent staffing constraints, indicating that local leadership work should be paired with policy‐level action.

## 1. Introduction

Nurses’ work environments are critical determinants of care quality, patient safety and workforce sustainability [1, 2]. Persistent staffing strain, communication breakdowns, constrained decision latitude and weak psychological safety continue to contribute to burnout and turnover, threatening the stability of healthcare delivery systems [3]. International policy and professional bodies increasingly frame these issues as system‐level performance risks rather than isolated managerial problems [4]. Leadership is consistently identified as one of the most modifiable organisational determinants of nurses’ work environments [5, 6]. Yet many leadership initiatives in nursing remain top‐down and training‐centred, often improving managerial knowledge without producing sustained improvements in frontline conditions [7, 8]. Recent nurse‐led paediatric programmes also demonstrate the clinical value of carefully structured nursing interventions [9], while evidence from digital neonatal care highlights the leadership, training, governance and workflow infrastructure needed for sustainable implementation [10].

This implementation gap has increased interest in participatory approaches that develop leadership practices with nurses, not only for nurses [11, 12]. This study examines a co‐created supportive leadership intervention implemented through PAR cycles in Saudi clinical settings. It investigates whether this approach is associated with measurable improvements in nurses’ work environments and workforce outcomes and explores candidate change pathways and contextual conditions associated with observed change. The Saudi context is particularly relevant because rapid health‐system transformation, workforce diversification and hierarchical organisational norms may shape both implementation and outcomes [13, 14].

Nurses’ work environments comprise the organisational and relational conditions under which care is delivered, including managerial support, quality of communication, participatory governance, resource facilitation and professional respect [15]. Favourable environments are associated with lower burnout, higher job satisfaction, stronger retention and improved safety climate, whereas unfavourable environments are associated with emotional exhaustion, disengagement and greater intention to leave [16–18]. Within this literature, leadership quality is a high‐impact factor. Supportive leadership includes both relational and structural functions. Relationally, it involves respectful communication, constructive feedback, recognition and psychological safety [19, 20]. Structurally, it includes barrier removal, procedural fairness, resource advocacy and facilitation of nurse participation in local decision‐making. This dual function is essential, as relational support alone is often insufficient when structural constraints persist [21–23]. Although prior studies report beneficial associations between supportive leadership and nurse outcomes, much of the evidence remains cross‐sectional, limiting inference about mechanisms, temporal ordering and implementation conditions [19, 20, 24]. Recent qualitative evidence from neonatal intensive care similarly shows how hierarchy and policy‐bound structures can constrain nurses’ voice and moral agency [25].

Co‐creation involves frontline nurses, nurse leaders and relevant stakeholders as partners in diagnosis, design, implementation and review. In complex clinical environments, where tacit norms and workflow constraints shape feasibility, co‐creation can improve contextual fit, ownership and implementation fidelity [26]. PAR offers a coherent methodology for operationalising co‐created leadership through iterative cycles of diagnosis, planning, action, reflection and adaptation. In nursing, PAR has shown value for engagement and practice relevance. However, leadership‐focused PAR studies often underspecify mechanisms, boundary conditions and the architecture of outcomes. Many reports that change occurred but provide a limited explanation of how the change was generated, under which conditions and why effects varied across settings [27, 28]. Recent nurse‐led photovoice research likewise illustrates how participatory methods can position children and family caregivers as active co‐producers of change rather than passive recipients [29].

The key gap is not whether leadership matters, but rather how co‐created supportive leadership, enacted through PAR, is associated with changes in nurses’ work environments and workforce outcomes through candidate proximal processes. Evidence rarely integrates proximal processes (e.g., psychological safety, voice, relational trust) with distal outcomes (e.g., burnout, job satisfaction and turnover intention) within one analytic model. Contextual contingencies such as staffing burden, hierarchy intensity and organisational readiness are also underexamined as moderators. Addressing this gap advances nursing management science from static association models toward pathway‐informed, implementation‐relevant evidence. It also offers practice and policy value by informing feasible strategies to strengthen nurses’ work environments and workforce stability under sustained operational pressure [30, 31].

### 1.1. Aim

To examine the processes, candidate change pathways and outcomes of a co‐created supportive leadership intervention implemented through iterative PAR cycles in clinical nursing settings in Saudi Arabia.

### 1.2. Research Questions

1. How is a co‐created supportive leadership intervention delivered through PAR associated with changes in nurses’ work‐environment conditions in participating clinical units?
2. Which candidate proximal processes and contextual conditions are associated with observed workforce outcome changes?

### 1.3. Objectives

1. Identify unit‐specific work‐environment barriers and leadership priorities through participatory baseline inquiry.
2. Codesign and implement context‐tailored supportive leadership actions across PAR cycles.
3. Assess longitudinal change in the Practice Environment Scale of the Nursing Work Index (PES‐NWI) composite and its five domains: nurse manager ability, leadership and support; nurse participation in hospital affairs; nursing foundations for quality of care; collegial nurse–physician relations; and staffing and resource adequacy.
4. Assess associated changes in four prespecified secondary workforce endpoints (personal and work‐related burnout, turnover intention and job satisfaction) and exploratory client‐related burnout.
5. Explore whether changes in psychological safety, perceived voice, relational trust and process‐level communication quality are consistent with candidate concurrent change pathways.
6. Explore interaction patterns involving staffing burden, hierarchy intensity and organisational readiness as potential contextual influences on pathway strength.

Nurses’ work environments are modifiable, but durable improvement requires participatory, context‐responsive leadership change that is feasible in routine care. Although supportive leadership is widely recognised, evidence remains limited regarding the candidate processes and pathways to real‐world implementation. This study addresses that limitation through a PAR‐based co‐creation approach that evaluates both process and outcomes.

### 1.4. Theoretical Framework

This investigation is guided by an integrated programme theory informed by supportive and transformational leadership concepts [32], structural empowerment theory [33] and PAR process logic [34] (Figure 1). PAR cycles (diagnose, codesign, act, reflect, adapt) function as the implementation engine, generating co‐created supportive leadership actions. Proposition 1: Co‐created leadership actions are expected to be associated with improvement in candidate proximal variables (psychological safety, perceived voice, relational trust and process‐level communication quality). Proposition 2: Improvements in candidate proximal variables are expected to be associated with more favourable scores on the five PES‐NWI domains: nurse manager ability, leadership and support; nurse participation in hospital affairs; nursing foundations for quality of care; collegial nurse–physician relations; and staffing and resource adequacy. Proposition 3: More favourable work‐environment outcomes are expected to be associated with lower personal, work‐related and client‐related burnout, lower turnover intention and higher job satisfaction. Proposition 4: Contextual factors may modify pathway strength (staffing burden and hierarchy intensity on earlier pathways; organisational readiness on downstream pathways). These propositions are directional and theory‐informed; they structure analysis without assuming fixed linear causality. Because the evaluation was uncontrolled, they were treated as a heuristic structure for exploratory concurrent change‐pathway analysis rather than formal causal hypotheses. Consistent with PAR epistemology, relationships were iteratively refined across cycles as emergent findings informed adaptation.

**FIGURE 1:**
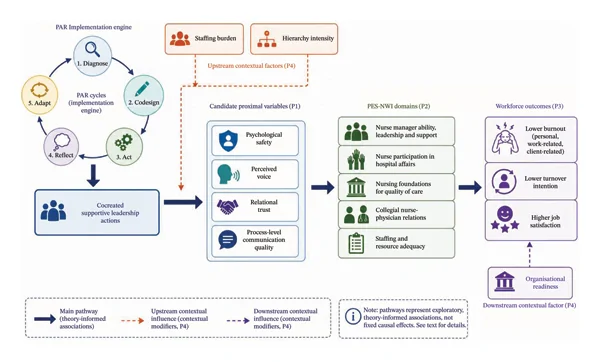
Integrated programme theory linking PAR‐based co‐created supportive leadership, candidate proximal variables, the five PES‐NWI domains, five workforce outcome variables and contextual influences. Note. Co‐created supportive leadership actions generated through participatory action research (PAR) cycles were expected to relate to candidate proximal variables (P1), the five practice environment scale of the nursing work index (PES‐NWI) domains (P2) and five workforce outcome variables (P3). Staffing burden and hierarchy intensity were positioned as upstream contextual influences and organisational readiness as a downstream influence (P4). Because the evaluation was uncontrolled, all pathways were treated as exploratory associations rather than causal effects. The figure was created for this manuscript and contains no third‐party logos or images.

## 2. Methods

### 2.1. Study Design

A multisite PAR design with an embedded uncontrolled single‐arm longitudinal mixed‐methods pre–post evaluation [34] was employed across five public hospitals in the Jouf region, Saudi Arabia. The study is framed primarily as an exploratory participatory implementation evaluation rather than as a mechanism‐confirming or causal intervention trial. PAR was selected because the intervention required iterative co‐creation and real‐time adaptation within routine clinical workflows, precluding protocol‐fixed experimental delivery. Implementation proceeded through three sequential PAR cycles at each site, each comprising diagnosis, codesign, action, reflection and adaptation. The quantitative strand assessed change in prespecified work‐environment and workforce outcomes at three measurement waves: baseline (T0, November 2024), midpoint (T1, during Cycle 2: March–June 2025) and endline (T2, October 2025). The qualitative strand examined implementation processes, candidate pathway experiences and contextual variation longitudinally across cycles. The study used an embedded longitudinal mixed‐methods design: Quantitative surveys were collected at three waves; qualitative and cycle‐process data were collected throughout implementation; and integration occurred during cycle review, cross‐strand analysis and final interpretation. Midpoint timing varied by site within the March–June window; categorical T1 estimates therefore average assessments made at different elapsed times and implementation stages. Reporting adhered to GRAMMS [35], COREQ [36] and published PAR reporting recommendations [37].

The completed implementation and evaluation timeline was as follows:

- T0—Baseline diagnosis and recruitment: November 2024
- Cycle 1—Codesign and initial action: December 2024–February 2025
- Cycle 2 + T1—Midpoint evaluation: March–June 2025
- Cycle 3—Final reflection and adaptation: July–September 2025
- T2—Final evaluation and study exit: October 2025
- Postimplementation analysis and manuscript preparation: November 2025–February 2026.

### 2.2. Setting

Five public hospitals in northern Saudi Arabia (Jouf region) were purposively selected to maximise variation in bed capacity (200–420 beds), service level (secondary and tertiary), speciality configuration and case‐mix acuity. All hospitals provided core inpatient and emergency services and were accredited by the Saudi Central Board for Accreditation of Healthcare Institutions (CBAHI) in accordance with its standards. The five sites belonged to the same regional healthcare directorate, ensuring comparable administrative governance while preserving the operational diversity necessary for cross‐site contextual analysis.

### 2.3. Participants and Eligibility Criteria

#### 2.3.1. Quantitative Cohort

Participants were registered nurses in direct clinical care roles. Inclusion criteria were as follows: (a) full‐time clinical appointment (≥ 36 h/week), (b) ≥ 1 year of continuous employment at a participating hospital, (c) Arabic proficiency sufficient for instrument completion and (d) provision of written informed consent. Nurses holding administrative‐only positions, those on temporary assignment (< 6 months) and those with planned extended leave during the data‐collection period were excluded.

#### 2.3.2. Qualitative Sample

Maximum‐variation purposive sampling ensured representation across hospitals, clinical specialities, years of experience and role strata (staff nurse, charge nurse, unit manager). Nurse managers who participated in codesign activities were included in the interview sample to capture implementation experiences from both leadership and frontline perspectives.

### 2.4. Sampling and Sample Size Justification

The eligible nursing population across the five hospitals was *N* = 1847, as verified from institutional human resources rosters. Stratified random sampling by hospital was applied for the quantitative cohort. The protocol’s a priori G∗Power Version 3.1 analysis [38] targeted within‐person change across three measurement waves, assuming a small‐to‐moderate standardised change, *α* = 0.05, power = 0.80 and correlation among repeated measures of *r* = 0.50; it yielded a minimum longitudinal analytic sample of *n* = 160. To retain at least 160 participants at endline under anticipated 20% attrition, the baseline enrolment target was *n* = 200 (160/0.80). A total of 240 eligible nurses were invited to reach that target after expected nonresponse; 200 enrolled and completed baseline assessment (response rate 83.3%), and 172 completed endline, remaining above the protocol’s within‐person analytic target. Hospital clustering was handled in the three‐level longitudinal models. The a priori calculation did not establish adequate power for hospital‐level variance components or cross‐level interactions; with only five hospital clusters, all interaction analyses were therefore designated exploratory and hypothesis‐generating.

For the qualitative component, four cross‐site focus groups (*n* = 36 staff and charge nurses; 8–10 per group) were conducted across the T1 and T2 cycle points, and six individual semistructured interviews with nurse managers were conducted at T2. All five hospitals were represented across the four groups; the six manager interviews included at least one manager from each hospital, with the two largest sites contributing an additional interview. The planned sample was completed and judged informationally adequate for the focused, framework‐informed questions. Combining hospitals within four groups supported cross‐site dialogue but constrained site‐level qualitative depth and may have inhibited critical disclosure; this limitation is acknowledged.

### 2.5. Intervention: PAR‐Based Cocreated Supportive Leadership

The intervention was delivered through three completed PAR cycles at each hospital over a 10‐month active implementation period within the 12‐month evaluation. Cycle duration ranged from 12 to 16 weeks, depending on site readiness and the local implementation trajectory. Each cycle comprised five phases:

1. Diagnosis: Participatory teams identified unit‐specific work‐environment barriers and leadership priorities using baseline survey summaries, routinely available unit‐performance indicators and structured team input sessions.
2. Codesign: Frontline nurses and nurse managers jointly developed unit‐tailored supportive leadership actions. Each action was documented with a named implementation owner, a defined timeline and a specified target candidate proximal variable (e.g., psychological safety, perceived voice, relational trust).
3. Action: Codesigned actions were implemented within existing unit workflows and available resources.
4. Reflection: Teams conducted standardised cycle‐end debriefs using a structured reflection guide that assessed feasibility, uptake, barriers, perceived effects and unintended consequences.
5. Adaptation: Based on reflection findings, actions were retained, modified or replaced before initiating the subsequent cycle.

Implementation fidelity was monitored through a structured cycle log and a PAR fidelity checklist completed at each site after each cycle. These tools documented adherence to core components of the PAR process while permitting context‐responsive tailoring. Facilitators completed 12 h of standardised preparation covering PAR principles, facilitation techniques and reflexive practice prior to implementation. Power asymmetry between participants and nurse leaders was mitigated through equal‐voice ground rules, rotating cofacilitation responsibilities and anonymous cycle‐end feedback mechanisms. A structured summary of the codesigned leadership actions, their target candidate proximal variables, participant composition and attendance, implementation dose and uptake, postreflection adaptations and fidelity across the three cycles is provided in Table 1.

**TABLE 1:** Summary of codesigned leadership actions, target candidate proximal variables, dose, adaptations and fidelity across PAR cycles (representative, aggregated across five sites).

PAR cycle (dates)	Representative codesigned actions and target candidate proximal variables	Participant composition/attendance	Implementation dose and uptake	Adaptations after reflection	Fidelity
Cycle 1 (Dec 2024–Feb 2025)	Equal‐voice huddle ground rules and structured preshift briefings (psychological safety); ‘you said–we did’ feedback boards (perceived voice); manager follow‐through commitments logged with named owners (relational trust)	Staff nurses, charge nurses and unit managers per site; mean attendance ≈80%–90% of invited PAR‐team members across the 5 sites	1 codesign + 1 reflection session per site; ≥ 1 action implemented per unit; uptake high for briefings, moderate for feedback boards	Feedback boards simplified; structured prompts added to link each action to its target mechanism	5/5 sites met all checklist criteria
Cycle 2 (Mar–Jun 2025; T1)	Corevised handover and scheduling routines responsive to nurse input (perceived voice); anonymous cycle‐end feedback channel (psychological safety); rotating cofacilitation (power‐asymmetry mitigation); communication‐quality standards for nurse–physician exchange (communication quality)	As above, manager participation increased; attendance ≈85%	1 codesign + 1 reflection per site; handover changes adopted in most units; nurse–physician standards variably adopted	Hierarchy‐sensitisation discussion added in high‐hierarchy units; one cycle extended at Hospital D	4/5 sites met all criteria; Hospital D met 5/6 (cofacilitation rotation partially implemented due to a scheduling conflict)
Cycle 3 (Jul–Sep 2025)	Consolidation and embedding of retained actions; local ownership of huddles and feedback loops; sustainability planning (organisational readiness)	As above, attendance ≈80%	1 reflection + adaptation per site; retained: briefings, follow‐through logs, anonymous feedback; abandoned: low‐uptake duplicate documentation	Retained actions institutionalised; few new actions introduced (consolidation phase)	5/5 sites met all checklist criteria

*Note:* Entries are representative descriptions aggregated across the five sites and derived from standardised cycle‐reflection templates and cycle logs (audit trail); site‐ and unit‐specific actions, attendance counts and dose indicators are retained in the study audit trail. Target labels identify the candidate proximal variable that each action was intended to influence; they do not establish a causal pathway.

Work‐environment outcomes were measured using the 31‐item PES‐NWI [39], Arabic version [40]. Workforce outcomes comprised burnout (Copenhagen Burnout Inventory [CBI]) [41], turnover intention (Turnover Intention Scale‐6 [TIS‐6]) [42] and job satisfaction (adapted Arabic Minnesota Satisfaction Questionnaire Short Form [MSQ‐SF]) [43]. Candidate proximal variables examined in exploratory change‐pathway models were adapted from Edmondson’s psychological safety scale [44], a perceived voice scale [45], an interpersonal trust scale [46] and communication‐quality items [47]. Contextual variables included the Organisational Readiness for Implementing Change (ORIC) scale [48], administrative nurse‐to‐patient ratios (staffing burden) and an adapted hierarchy/power‐distance intensity scale [49]. Instruments without directly transferable Arabic psychometric evidence underwent a standardised seven‐step adaptation and psychometric evaluation protocol; preliminary within‐sample measurement checks met prespecified thresholds (*α* ≥ 0.70; Supporting Table S1). A discriminant‐validity check supported measurement distinction between process‐level communication quality and the PES‐NWI collegial nurse–physician relations domain (*r* = 0.47; Δχ^2^ = 34.2, *p* < 0.001; Supporting Table S3).

### 2.6. Variables and Data‐Collection Instruments

All study variables were mapped a priori to the programme‐theory sequence: co‐created leadership actions ⟶ candidate proximal variables ⟶ PES‐NWI work‐environment outcomes ⟶ workforce outcomes, with contextual variables positioned at prespecified segments. Supporting Table S1 summarises instrument sources, adaptation, scoring and preliminary psychometric properties.

#### 2.6.1. Work‐Environment Outcomes

Work‐environment dimensions were measured using the 31‐item PES‐NWI [39], which covers five domains: nurse manager ability, leadership and support; nurse participation in hospital affairs; nursing foundations for quality of care; collegial nurse–physician relations; and staffing and resource adequacy. The Arabic PES‐NWI version [40] was used. Because Arabic psychometric performance may vary across nursing contexts, reliability and construct performance were re‐evaluated in the present sample before inferential modelling. Item wording was harmonised for regional linguistic clarity without structural modification (no item addition, deletion or domain respecification). Baseline internal consistency and dimensionality results for retained PES‐NWI domains are reported in the Results section and in Supporting Table S1.

#### 2.6.2. Workforce Outcomes (Secondary and Exploratory)

Burnout was measured using the CBI [41], which comprises personal, work‐related and client‐related subscales. Personal and work‐related burnout were prespecified secondary endpoints; client‐related burnout was analysed as an exploratory workforce outcome. The Arabic‐language form was used, and subscale reliability was assessed in the present sample before modelling. Turnover intention, a prespecified secondary endpoint, was measured using the six‐item TIS [42]; higher scores indicated stronger turnover intention.

Job satisfaction was measured using a 14‐item study‐adapted Arabic form derived from the MSQ‐SF [43]. Items were selected from relevant MSQ‐SF domains and contextually refined for nursing applicability. Because no directly transferable Arabic nursing psychometric evidence existed for this exact population, the instrument underwent the full seven‐step adaptation and psychometric evaluation workflow described in the Translation, Adaptation and Psychometric Evaluation section.

#### 2.6.3. Candidate Proximal Variables

Four candidate proximal variables were examined in the exploratory change‐pathway analyses. Psychological safety was measured with a six‐item study‐adapted form derived from Edmondson’s Team Psychological Safety scale (score range 6–42). Perceived openness to voice was measured with a five‐item study‐adapted form derived from the LePine–Van Dyne voice‐behaviour scale. Relational trust used eight adapted affect‐ and cognition‐based items, and process‐level interpersonal communication quality used seven adapted items derived from the ICU Nurse–Physician Questionnaire communication subscale and healthcare‐team interaction items. These study‐adapted measures underwent the full translation, adaptation and preliminary within‐sample psychometric workflow described below (Supporting Table S1).

#### 2.6.4. Contextual Moderators

Organisational readiness for change was measured using the ORIC scale [48], which assesses change commitment and change efficacy. Where a fully validated Arabic form specific to this setting was unavailable, the seven‐step adaptation procedures (see the Translation, Adaptation and Psychometric Evaluation section) were applied. Staffing burden was operationalised as the unit‐level nurse‐to‐patient ratio at each measurement wave, extracted from hospital administrative records. Higher ratios indicated greater patient load per nurse. Hierarchy intensity was measured using a structured scale adapted from established items assessing organisational power distance and workplace hierarchy [49].

#### 2.6.5. Qualitative Data Collection Tools

Four qualitative instruments were used: (a) a semistructured focus‐group guide for staff and charge nurses, (b) a semistructured interview guide for nurse managers, (c) a standardised cycle‐reflection template, and (d) a field‐note and cycle‐log protocol. The guides were developed in Arabic, reviewed by three qualitative nursing researchers, piloted with noncohort participants and version‐controlled. Sessions were conducted in Arabic by trained facilitators, and a second team member checked transcripts against the source audio. Full development and quality‐assurance procedures are reported in Supporting Table S2.

#### 2.6.6. Prespecified Distinction Between Process Communication and the PES‐NWI Comparator

Communication was measured at two distinct levels: process‐level interpersonal communication quality (candidate proximal variable) and the PES‐NWI collegial nurse–physician relations domain (work‐environment outcome). At baseline, the two scores correlated at *r* = 0.47. A two‐factor model fit better than a one‐factor model, Δχ^2^(1) = 34.2, *p* < 0.001 (two‐factor CFI = 0.94, RMSEA = 0.06; one‐factor CFI = 0.81, RMSEA = 0.11), supporting measurement distinction in this sample without establishing causal pathway separation (Supporting Table S3).

### 2.7. Translation, Adaptation and Psychometric Evaluation

For constructs without directly transferable Arabic psychometric evidence in the target population (job satisfaction, psychological safety, perceived voice, relational trust, organisational readiness, hierarchy intensity and process‐level communication quality), a standardised seven‐step adaptation protocol was completed before inferential modelling (source mapping, dual forward translation, reconciliation, blind back‐translation, expert review, cognitive debriefing/pilot testing and baseline psychometric testing). Instrument‐level outcomes of this process, including removed items, refinements, Cronbach’s *α*, dimensionality and retention decisions, are summarised in Supporting Table S1. Prespecified adequacy thresholds were *α* ≥ 0.70, primary factor loadings ≥ 0.40 and interconstruct correlations < 0.85 for discriminant‐validity support. The critical discriminant test for the communication constructs (process‐level candidate proximal variable vs PES‐NWI collegial nurse–physician relations outcome) is additionally reported in Supporting Table S3.

### 2.8. Data‐Collection Procedure

Data were collected at three scheduled waves (T0, T1 and T2) by 10 trained research assistants who were independent of PAR facilitation. All assistants completed standardised training in participant recruitment, informed consent, neutral instrument administration, confidentiality and data quality control procedures.

The prespecified procedure was implemented as follows:

1. Site coordinators generated eligibility lists from institutional HR rosters at each wave.
2. Eligible nurses were approached, screened against inclusion/exclusion criteria and consented.
3. Each participant was assigned a unique anonymised identifier to enable longitudinal linkage across T0, T1 and T2.
4. Questionnaires were administered in supervised sessions during protected nonclinical time windows to minimise workflow disruption.
5. Administrative staffing indicators were extracted for the corresponding wave periods.
6. Focus groups and interviews were conducted at prespecified time points, audio‐recorded with consent, transcribed verbatim, de‐identified and verified against the recordings. Coding was conducted in Arabic; quotations selected for publication were translated into English and independently reviewed through back‐translation.
7. Cycle logs, structured reflection templates and field notes were completed continuously throughout implementation.
8. After each wave, quality checks were performed for completeness, range plausibility, identifier consistency and missingness patterns.
9. Final locked analysis datasets were prepared after full reconciliation of survey, administrative and qualitative data sources.

### 2.9. Data Analysis

#### 2.9.1. Quantitative Analysis

Descriptive statistics were used to summarise participant demographics and site characteristics across measurement waves. Longitudinal change across T0–T2 was estimated using three‐level mixed‐effects models with the following:

• Level 1: repeated measures (time: T0, T1, T2) nested within participants,
• Level 2: participant‐level random intercepts
• Level 3: hospital‐level random intercepts.

Time was modelled as a categorical factor (T0 reference; T1 and T2 contrasts), avoiding an assumption of linear change. Models used an unstructured covariance matrix for repeated measurements when its parameters could be estimated; if they could not be estimated, a compound‐symmetry covariance matrix was used. Prespecified fixed covariates were age, gender and years of clinical experience; hospital was represented by the Level 3 random intercept. Hospital‐level random slopes for time were tested using likelihood‐ratio tests and information criteria and retained where fit improved, but the five‐site sample provides limited support for stable random‐slope or between‐hospital inference. Fixed‐effect estimates were reported with 95% confidence intervals. The standardised T0–T2 mean change (d) was calculated as the model‐estimated T0–T2 change divided by the pooled observed T0 and T2 standard deviation, SDpooled = √[(SDT0^2^ + SDT2^2^)/2].

Outcomes were organised into prespecified analytic tiers. The longitudinal PES‐NWI composite was the primary outcome. Personal burnout, work‐related burnout, turnover intention and job satisfaction were prespecified secondary endpoints; the five PES‐NWI domain scores and client‐related burnout were exploratory outcomes. The 20 concurrent change‐pathway products and nine interaction tests were also exploratory. Benjamini–Hochberg false‐discovery‐rate correction was applied to the nine interactions. The 20 change‐pathway products and domain‐specific PES‐NWI estimates were not multiplicity‐adjusted and were interpreted from effect sizes and confidence intervals rather than as confirmatory tests.

Because all hospitals received the programme and exposure was indexed only by measurement occasion, the analysis did not estimate causal intervention mediation. Exploratory product‐of‐coefficients models linked concurrent T0–T2 change in each candidate proximal variable with change in each PES‐NWI domain, conditional on baseline proximal and outcome scores, age, gender, clinical experience and a hospital random intercept. We first created 20 imputed datasets. We fitted the models in each dataset and then drew 5,000 participant‐level bootstrap resamples within hospitals from each dataset; imputation‐specific point estimates were averaged; and the resampled product estimates were combined to form pooled bias‐corrected 95% confidence intervals reflecting within‐ and between‐imputation variation. The larger proximal‐variable gains at T0–T1 were examined only as a temporal pattern. Because proximal and outcome changes were concurrent and programme dose was not measured, the products are candidate change‐pathway associations and do not establish temporal mediation.

Moderation was explored using prespecified interaction terms entered with their main effects: candidate proximal variable × staffing burden, candidate proximal variable × hierarchy intensity and work environment × organisational readiness. Staffing burden was a unit‐level administrative variable; hierarchy intensity and readiness were baseline individual‐perception measures. Given only five hospitals, all interaction estimates were treated as hypothesis‐generating and reported with confidence intervals, unadjusted *p* values and FDR‐adjusted *q* values. Because conditional simple slopes were not reported, nominal interaction coefficients were not interpreted as confirmed attenuation or amplification.

Missing data were addressed using multiple imputation by fully conditional specification (*m* = 20 imputations). The imputation model included all analysis variables (outcomes, candidate proximal variables, contextual variables and covariates), the hospital indicator to reflect clustering and auxiliary variables associated with missingness; continuous variables were imputed by predictive mean matching and categorical variables by logistic models. Convergence was checked through trace plots, and imputed distributions were compared with observed distributions. Estimates were pooled using Rubin’s rules. Little’s MCAR test and completer‐versus‐noncompleter comparisons did not provide evidence against MCAR and were compatible with, but did not prove, the missing‐at‐random assumption used for imputation. Complete‐case analyses were conducted as a sensitivity check (Supporting Table S4).

All quantitative analyses were performed using IBM SPSS Statistics Version 27.0 (IBM Corp., Armonk, NY, USA). To reduce interpretive bias arising from the principal investigator’s dual role, model specifications and statistical outputs were independently reviewed by a quantitative‐methods adviser who was not involved in PAR facilitation, and qualitative coding decisions were examined through an independent audit (see the Reflexivity section). Given the uncontrolled PAR pre–post design without random assignment, all findings are interpreted as association‐ and pathway‐consistent rather than causal; causal verbs are avoided in favour of associational language.

#### 2.9.2. Qualitative Analysis

Qualitative data were analysed using reflexive thematic analysis with combined deductive–inductive coding [50]. Deductive codes were informed by the study framework, while inductive coding captured unanticipated meanings. Analysis focused on implementation dynamics, candidate pathway experiences and contextual variation. Trustworthiness was supported through triangulation, analytic memos, an audit trail, participant reflection on synthesised interpretations and review of a purposively selected 20% of transcripts, memos and the evolving thematic map by a qualitative researcher not involved in facilitation. Differences in interpretation were discussed as reflexive prompts that refined theme boundaries; no inter‐rater reliability target was applied.

#### 2.9.3. Mixed‐Methods Integration

The study used an embedded longitudinal mixed‐methods integration strategy. Quantitative surveys were collected at three waves; qualitative and cycle‐process data informed successive adaptations; and integration occurred at cycle review, cross‐strand analysis and final interpretation through joint displays. Meta‐inferences assessed convergence, complementarity and divergence without treating qualitative findings as statistical validation.

### 2.10. Rigour and Trustworthiness

Rigour in the quantitative strand was strengthened by standardised data‐collection procedures, prespecified analytic models, preliminary within‐sample measurement checks prior to inferential testing, model‐diagnostic checks and sensitivity analyses (including complete‐case comparisons with imputed models). Trustworthiness in the qualitative strand was established by addressing credibility, dependability, confirmability and transferability through triangulation across data sources, maintaining a documented audit trail, reflexive memoing, an independent audit of a subset of coded transcripts, participant review of synthesised interpretations, and thick contextual description of study settings and implementation conditions [51–53]. Mixed‐methods rigour was supported by a prespecified integration protocol, framework‐aligned joint displays and transparent meta‐inference development informed by convergence, complementarity and divergence across strands.

### 2.11. Reflexivity

Given the principal investigator’s dual role as researcher and PAR facilitator, reflexivity was embedded across all study phases. A structured reflexive journal documented positionality, evolving assumptions, interactional dynamics, key methodological decisions and their rationale. After each PAR cycle, formal debriefing sessions with research‐team advisers critically interrogated emergent interpretations and challenged potential overattribution to facilitator perspectives. An independent qualitative audit of a subset of transcripts and coding outputs provided an additional external check on facilitator‐linked interpretive bias.

### 2.12. Ethical Considerations

Ethical approval was obtained from the Jouf University Institutional Review Board (Protocol No. 7603) and from the ethics committees of all participating hospitals prior to recruitment. Written informed consent was obtained from all participants before data collection. Participation was voluntary, and participants were informed of their right to withdraw at any stage without occupational consequences. Given the participatory design, PAR‐specific safeguards were implemented: Participants received explicit information about their dual participant/coresearcher role and related rights; confidentiality agreements were established at the start of all participatory sessions; reflection‐session content was anonymised prior to analysis; cross‐site sharing of identifiable information was prohibited; and all study data were stored on secure, encrypted, password‐protected systems with access restricted to authorised research‐team members.

## 3. Results

### 3.1. Participant Flow and Retention

Of 1847 eligible registered nurses, 240 were invited and 200 enrolled (83.3%). Retention was 93.0% at T1 (186/200) and 86.0% at T2 (172/200). Endline attrition comprised interhospital transfer (*n* = 11), resignation (*n* = 8), extended leave (*n* = 5) and voluntary withdrawal (*n* = 4); thus, 19 of 28 losses involved transfer or resignation, and eight participants (4.0% of the baseline cohort) resigned during follow‐up. Little’s MCAR test was nonsignificant, *χ* ^2^ = 42.61, df = 38, *p* = 0.28, and baseline comparisons detected no differences (all *p* > 0.10). These diagnostics do not establish MCAR or MAR. Multiple imputation therefore rests on an explicit MAR assumption, and residual bias from informative departure cannot be excluded, particularly for turnover‐related outcomes.

All 15 planned PAR cycle debriefs (3 cycles × 5 hospitals) were completed. Cycle‐log review showed that 14 of 15 sessions met all PAR fidelity checklist criteria; one Cycle 2 session at Hospital D met 5 of 6 criteria because cofacilitation rotation was partially implemented owing to a scheduling conflict. Qualitative data collection proceeded as planned: Four focus groups (*n* = 36 nurses) were conducted across T1 and T2 cycle points, and six semistructured manager interviews were completed at T2.

### 3.2. Baseline Characteristics

Table 2 presents participant demographic and professional characteristics at baseline. The sample was predominantly female (82.0%), with a mean age of 31.4 years (SD = 5.7). The majority held a Bachelor of Science in Nursing (67.0%), and the mean clinical experience was 7.2 years (SD = 4.3). Non‐Saudi nurses accounted for 66.0% of the sample, reflecting the regional composition of the nursing workforce. Participants were distributed across medical (24.0%), surgical (21.0%), critical care (18.0%), paediatric (14.0%), emergency (13.0%) and other specialities (10.0%). Hospital‐level distributions were proportional to the size of the eligible workforce at each site. No statistically significant baseline differences were observed across hospitals for primary or secondary outcomes (Kruskal–Wallis tests, all *p* > 0.05).

**TABLE 2:** Participant demographic and professional characteristics at baseline (*N* = 200).

Characteristic	Total sample (*N* = 200)
Demographic characteristics	
Age, years, *M* (SD)	31.4 (5.7)
Gender, *n* (%)	
Female	164 (82.0)
Male	36 (18.0)
Nationality, *n* (%)	
Saudi	68 (34.0)
Non‐Saudi	132 (66.0)
Marital status, *n* (%)	
Single	72 (36.0)
Married	118 (59.0)
Divorced/Widowed	10 (5.0)
Professional characteristics	
Highest nursing qualification, *n* (%)	
Nursing diploma	38 (19.0)
Bachelor of Science in Nursing	134 (67.0)
Master’s degree or above	28 (14.0)
Total clinical experience, years, *M* (SD)	7.2 (4.3)
Experience at current hospital, years, *M* (SD)	4.6 (3.1)
Current role, *n* (%)	
Staff nurse	160 (80.0)
Charge nurse	28 (14.0)
Unit manager	12 (6.0)
Employment type, *n* (%)	
Permanent	176 (88.0)
Contract	24 (12.0)
Clinical unit, *n* (%)	
Medical	48 (24.0)
Surgical	42 (21.0)
Intensive care	36 (18.0)
Paediatric	28 (14.0)
Emergency	26 (13.0)
Other (obstetric, oncology, outpatient)	20 (10.0)
Hospital distribution, *n* (%)	
Hospital A (tertiary, 420 beds)	44 (22.0)
Hospital B (tertiary, 380 beds)	42 (21.0)
Hospital C (secondary, 300 beds)	40 (20.0)
Hospital D (secondary, 250 beds)	38 (19.0)
Hospital E (secondary, 200 beds)	36 (18.0)

*Note: M* = mean; Percentages may not sum to 100 due to rounding. Non‐Saudi nationalities included Filipino (34.0%), Indian (16.5%), Malaysian (8.0%), Jordanian (4.5%) and other (3.0%).

Abbreviation: SD = standard deviation.

### 3.3. Instrument Reliability

All measures met the prespecified preliminary within‐sample reliability/loading thresholds at baseline (Cronbach’s *α* ≥ 0.70). The PES‐NWI demonstrated overall *α* = 0.91 (domains 0.79–0.88); CBI domains ranged from 0.82 to 0.87; TIS‐6 *α* = 0.83; and study‐adapted measures ranged from 0.77 to 0.89. Process‐level communication quality correlated *r* = 0.47 with the PES‐NWI collegial nurse–physician relations domain; a two‐factor model fit better than a one‐factor model, Δχ^2^(1) = 34.2, *p* < 0.001 (CFI 0.94 vs 0.81; RMSEA 0.06 vs 0.11), supporting measurement distinction in this sample (Supporting Tables S1 and S3).

### 3.4. Descriptive Statistics Across Measurement Waves

Table 3 presents observed means and standard deviations across waves. Scores shifted in a favourable direction for all outcomes, although model‐based inference varied by outcome and analytic tier. Manager support and nurse participation showed the largest observed work‐environment gains. Job satisfaction increased, while personal burnout and turnover intention decreased. All four candidate proximal‐variable scores increased from T0 to T2, with the largest observed gains in psychological safety and perceived voice.

**TABLE 3:** Descriptive statistics for study variables across measurement waves.

Variable	Scale range	T0 (baseline) *n* = 200 *M* (SD)	T1 (midpoint) *n* = 186 *M* (SD)	T2 (month 12) *n* = 172 *M* (SD)
Work‐environment outcomes (PES‐NWI domains)				
PES‐NWI composite	1.00–4.00	2.47 (0.51)	2.55 (0.50)	2.65 (0.48)
Nurse manager ability and support	1.00–4.00	2.51 (0.57)	2.63 (0.55)	2.78 (0.53)
Nurse participation in hospital affairs	1.00–4.00	2.36 (0.55)	2.47 (0.53)	2.59 (0.51)
Nursing foundations for quality of care	1.00–4.00	2.58 (0.49)	2.66 (0.48)	2.74 (0.46)
Collegial nurse–physician relations	1.00–4.00	2.53 (0.53)	2.60 (0.52)	2.68 (0.50)
Staffing and resource adequacy	1.00–4.00	2.37 (0.59)	2.43 (0.58)	2.50 (0.56)
Workforce outcomes				
CBI—personal burnout	0–100	57.8 (16.2)	52.4 (15.6)	50.3 (15.1)
CBI—work‐related burnout	0–100	53.4 (17.0)	49.1 (16.4)	46.8 (16.0)
CBI—client‐related burnout	0–100	41.6 (18.4)	39.2 (17.8)	37.8 (17.4)
Turnover intention (TIS‐6)	6–30	19.2 (4.7)	18.1 (4.6)	17.1 (4.4)
Job satisfaction (adapted MSQ‐SF)	14–70	42.1 (9.2)	44.6 (8.8)	46.4 (8.5)
Candidate proximal variables				
Psychological safety	6–42	24.1 (5.4)	27.2 (5.1)	27.8 (4.9)
Perceived voice	5–35	18.3 (4.3)	20.6 (4.1)	21.2 (3.9)
Relational trust	8–56	31.2 (7.8)	34.5 (7.4)	35.6 (7.1)
Process‐level communication quality	7–35	20.1 (4.6)	22.3 (4.4)	23.0 (4.2)
Contextual variables				
Organisational readiness (ORIC)	12–60	37.8 (8.2)	—	—
Staffing burden (nurse‐to‐patient ratio)	Continuous	1:5.8	1:5.6	1:5.5
Hierarchy intensity	5–25	17.1 (3.5)	—	—

*Note: M* = mean; PES‐NWI = Practice Environment Scale of the Nursing Work Index; ORIC = Organisational Readiness for Implementing Change. Organisational readiness and hierarchy intensity were measured at baseline for moderation analyses and were not reassessed longitudinally; dashes indicate that they were not measured. Staffing burden was extracted administratively at each wave; displayed nurse‐to‐patient ratios are descriptive wave summaries across participating units, with larger values indicating more patients per nurse. T1 represents the midpoint evaluation conducted during Cycle 2 (March–June 2025), and T2 represents the final evaluation conducted in October 2025. T1 and T2 values are observed means; model‐based estimates adjusted for clustering and missing data are reported in Table 4.

Abbreviations: CBI = Copenhagen Burnout Inventory, MSQ‐SF = Minnesota Satisfaction Questionnaire—Short Form, SD = standard deviation, TIS‐6 = Turnover Intention Scale‐6.

### 3.5. Longitudinal Change in Work‐Environment and Workforce Outcomes

Table 4 presents mixed‐effects estimates for change from baseline to midpoint and endline. Hospital‐level ICC point estimates ranged from 0.02 to 0.05; with only five sites, these variance estimates are imprecise and do not establish stable between‐hospital heterogeneity. For the primary PES‐NWI composite, T0–T2 change was *β* = 0.18 (95% CI [0.10, 0.26], *p* < 0.001, *d* = 0.36). Exploratory domain estimates were largest for nurse manager ability, leadership and support (*β* = 0.27, 95% CI [0.17, 0.37], *d* = 0.49) and nurse participation (*β* = 0.23, 95% CI [0.13, 0.33], *d* = 0.43). Collegial nurse–physician relations changed less (*β* = 0.15, 95% CI [0.05, 0.25], *d* = 0.29), and staffing/resource adequacy had the smallest, borderline estimate (*β* = 0.13, 95% CI [0.01, 0.25], *p* = 0.038, *d* = 0.23). Domain estimates were unadjusted and exploratory.

**TABLE 4:** Three‐level mixed‐effects model estimates: Change in work‐environment and workforce outcomes (*N* = 200).

Outcome	T0 ⟶ T1			T0 ⟶ T2			Cohen’s *d*	Hospital ICC
	*β* (SE)	95% CI	*p*	*β* (SE)	95% CI	*p*		
Work‐environment outcomes (PES‐NWI)								
Composite score	0.08 (0.03)	[0.02, 0.14]	0.008	0.18 (0.04)	[0.10, 0.26]	< 0.001	0.36	0.03
Manager ability and support	0.12 (0.04)	[0.04, 0.20]	0.002	0.27 (0.05)	[0.17, 0.37]	< 0.001	0.49	0.04
Nurse participation	0.11 (0.04)	[0.03, 0.19]	0.005	0.23 (0.05)	[0.13, 0.33]	< 0.001	0.43	0.03
Foundations for quality	0.08 (0.03)	[0.02, 0.14]	0.012	0.16 (0.04)	[0.08, 0.24]	< 0.001	0.34	0.02
Nurse–physician relations	0.07 (0.04)	[−0.01, 0.15]	0.072	0.15 (0.05)	[0.05, 0.25]	0.004	0.29	0.03
Staffing and resource adequacy	0.06 (0.04)	[−0.02, 0.14]	0.132	0.13 (0.06)	[0.01, 0.25]	0.038	0.23	0.05
Workforce outcomes								
Personal burnout (CBI)	−5.4 (1.4)	[−8.1, −2.7]	< 0.001	−7.5 (1.6)	[−10.6, −4.4]	< 0.001	−0.48	0.03
Work‐related burnout	−4.3 (1.5)	[−7.2, −1.4]	0.004	−6.6 (1.7)	[−9.9, −3.3]	< 0.001	−0.40	0.04
Client‐related burnout	−2.4 (1.6)	[−5.5, 0.7]	0.132	−3.8 (2.0)	[−7.7, 0.1]	0.062	−0.21	0.02
Turnover intention‐6 (TIS‐6)	−1.1 (0.4)	[−1.9, −0.3]	0.008	−2.1 (0.4)	[−2.9, −1.3]	< 0.001	−0.46	0.04
Job satisfaction	2.5 (0.8)	[0.9, 4.1]	0.002	4.3 (0.9)	[2.5, 6.1]	< 0.001	0.49	0.03

*Note: β* = unstandardised fixed‐effect estimate for the time contrast (T0 reference); *d* = model‐estimated T0–T2 change divided by the pooled observed T0 and T2 standard deviation, SDpooled = √[(SDT0^2^ + SDT2^2^)/2]; ICC = hospital‐level intraclass correlation. Negative *d* values denote favourable reductions. Models included participant‐ and hospital‐level random intercepts; hospital time slopes were retained for manager ability/support and turnover intention after likelihood‐ratio testing, but random‐slope inference is imprecise with five sites. Estimates use 20 imputations. Complete‐case estimates were broadly concordant; staffing/resource adequacy crossed the conventional .05 threshold (*p* = 0.038 imputed vs .054 complete case; Supporting Table S4).

Abbreviations: CI = confidence interval, SE = standard error.

Among the four prespecified secondary endpoints, personal burnout decreased (*β* = −7.5, *d* = −0.48, *p* < 0.001), work‐related burnout decreased (*β* = −6.6, *d* = −0.40, *p* < 0.001), turnover intention decreased (*β* = −2.1, *d* = −0.46, *p* < 0.001), and job satisfaction increased (*β* = 4.3, *d* = 0.49, *p* < 0.001). Exploratory client‐related burnout showed a smaller, nonsignificant reduction (*β* = −3.8, *d* = −0.21, *p* = 0.062). Complete‐case estimates were directionally and quantitatively similar; 10 of 11 T0–T2 tests retained the same conventional *p* < 0.05 classification. Staffing/resource adequacy attenuated from *β* = 0.13 (95% CI [0.01, 0.25], *p* = 0.038, *d* = 0.23) in the imputed analysis to *β* = 0.12 (95% CI [0.00, 0.24] after rounding, *p* = 0.054, *d* = 0.21) in complete cases, making this borderline exploratory inference sensitive to missing‐data handling (Supporting Table S4).

### 3.6. Change in Candidate Proximal Variables

All four candidate proximal‐variable scores increased from T0 to T2 (each nominal *p* < 0.001; no multiplicity adjustment). Psychological safety showed the largest standardised change (*d* = 0.72), followed by perceived voice (*d* = 0.71), process‐level communication quality (*d* = 0.66) and relational trust (*d* = 0.59). The largest gains occurred between T0 and T1, with smaller increments between T1 and T2. This timing is consistent with, but does not establish, earlier change in candidate proximal variables followed by consolidation in the final cycle.

### 3.7. Exploratory Concurrent Change‐Pathway Analysis

Table 5 presents the exploratory bootstrap product‐of‐coefficient estimates linking concurrent change in four candidate proximal variables with change in five PES‐NWI domains. Thirteen of 20 bias‐corrected 95% confidence intervals excluded zero. Because these products were unadjusted for multiplicity, used concurrent changes and came from an uncontrolled evaluation, they are descriptive candidate change‐pathway associations rather than indirect causal effects or confirmed mediation.

**TABLE 5:** Exploratory bootstrap product‐of‐coefficients estimates for concurrent change pathways (5000 resamples per imputed dataset).

Candidate proximal variable	Work‐environment outcome	Product estimate (*β*)	95% bias‐corrected CI	Bootstrap 95% CI result
Psychological safety	Manager ability and support	0.08	[0.04, 0.14]	Excludes zero
	Nurse participation	0.07	[0.03, 0.12]	Excludes zero
	Foundations for quality	0.05	[0.01, 0.10]	Excludes zero
	Nurse–physician relations	0.04	[−0.01, 0.09]	Includes zero
	Staffing and resources	0.02	[−0.02, 0.07]	Includes zero

Perceived voice	Manager ability and support	0.07	[0.03, 0.13]	Excludes zero
	Nurse participation	0.09	[0.04, 0.15]	Excludes zero
	Foundations for quality	0.04	[0.01, 0.09]	Excludes zero
	Nurse–physician relations	0.03	[−0.01, 0.08]	Includes zero
	Staffing and resources	0.01	[−0.03, 0.06]	Includes zero

Relational trust	Manager ability and support	0.06	[0.02, 0.12]	Excludes zero
	Nurse participation	0.05	[0.01, 0.10]	Excludes zero
	Foundations for quality	0.03	[−0.01, 0.08]	Includes zero
	Nurse–physician relations	0.06	[0.02, 0.11]	Excludes zero
	Staffing and resources	0.02	[−0.02, 0.07]	Includes zero

Process‐level communication quality	Manager ability and support	0.05	[0.01, 0.10]	Excludes zero
	Nurse participation	0.04	[0.01, 0.09]	Excludes zero
	Foundations for quality	0.04	[0.01, 0.09]	Excludes zero
	Nurse–physician relations	0.05	[0.01, 0.10]	Excludes zero
	Staffing and resources	0.01	[−0.03, 0.06]	Includes zero

*Note:* The workflow was impute then bootstrap: Models were fitted in each of 20 imputed datasets; 5000 participant‐level resamples were drawn within hospital per dataset; imputation‐specific point estimates were averaged; and resampled product estimates were combined to obtain pooled bias‐corrected 95% CIs. Products link concurrent T0–T2 changes; adjust for baseline proximal and outcome scores, age, gender and clinical experience; and include a hospital random intercept. ‘Excludes zero’ does not denote mediation. The 20 products were exploratory and unadjusted; 13 intervals excluded zero, and none involving staffing/resource adequacy did so.

Thirteen of the 20 exploratory intervals excluded zero. Psychological safety products excluded zero for manager ability/support (*β* = 0.08, 95% CI [0.04, 0.14]), nurse participation (*β* = 0.07, 95% CI [0.03, 0.12]) and foundations for quality (*β* = 0.05, 95% CI [0.01, 0.10]). Perceived voice products excluded zero for manager ability/support (*β* = 0.07, 95% CI [0.03, 0.13]), nurse participation (*β* = 0.09, 95% CI [0.04, 0.15]) and foundations for quality (*β* = 0.04, 95% CI [0.01, 0.09]). Relational trust products excluded zero for manager ability/support (*β* = 0.06, 95% CI [0.02, 0.12]), nurse participation (*β* = 0.05, 95% CI [0.01, 0.10]) and nurse–physician relations (*β* = 0.06, 95% CI [0.02, 0.11]). Process‐level communication products excluded zero for manager ability/support (*β* = 0.05, 95% CI [0.01, 0.10]), nurse participation (*β* = 0.04, 95% CI [0.01, 0.09]), foundations for quality (*β* = 0.04, 95% CI [0.01, 0.09]) and nurse–physician relations (*β* = 0.05, 95% CI [0.01, 0.10]). No staffing/resource‐adequacy interval excluded zero.

### 3.8. Moderation Analysis

Table 6 presents nine prespecified exploratory interaction tests. Four met the nominal pre‐FDR *p* < 0.05 threshold: staffing burden × psychological safety (*β* = −0.04, *p* = 0.012, *q* = 0.054), hierarchy intensity × perceived voice (*β* = −0.03, *p* = 0.024, *q* = 0.072), readiness × work environment for personal burnout (*β* = 0.05, *p* = 0.008, *q* = 0.054) and readiness × work environment for job satisfaction (*β* = 0.04, *p* = 0.046, *q* = 0.103). None survived Benjamini–Hochberg correction (smallest *q* = 0.054). Readiness × turnover intention was not nominally significant (*p* = 0.098, *q* = 0.176). Because conditional simple slopes were unavailable and only five sites contributed data, these coefficients are hypothesis‐generating signals whose practical directions cannot be labelled amplification or attenuation.

**TABLE 6:** Exploratory interaction (moderation) analysis of contextual factors and framework pathways.

Moderator	Tested pathway	Interaction *β* (SE)	95% CI	*p* (BH *q*)	Interpretation
Staffing burden (nurse‐to‐patient ratio)	Psych. safety ⟶ work env.	−0.04 (0.02)	[−0.07, −0.01]	0.012 (0.054)	Nominal negative signal; NS after FDR (*q* = 0.054)
	Perceived voice ⟶ work env.	−0.02 (0.02)	[−0.06, 0.02]	0.284 (0.319)	No nominal signal
	Relational trust ⟶ work env.	−0.01 (0.02)	[−0.05, 0.03]	0.518 (0.518)	No nominal signal

Hierarchy intensity	Psych. safety ⟶ work env.	−0.02 (0.02)	[−0.05, 0.01]	0.164 (0.211)	No nominal signal
	Perceived voice ⟶ work env.	−0.03 (0.01)	[−0.06, −0.01]	0.024 (0.072)	Nominal negative signal; NS after FDR (*q* = 0.072)
	Comm. Quality ⟶ work env.	−0.02 (0.01)	[−0.05, 0.01]	0.142 (0.211)	No nominal signal

Organisational readiness (ORIC)	Work env. ⟶ personal burnout	0.05 (0.02)	[0.01, 0.09]	0.008 (0.054)	Nominal positive signal; NS after FDR (*q* = 0.054)
	Work env. ⟶ job satisfaction	0.04 (0.02)	[0.00, 0.08]	0.046 (0.103)	Nominal *p* < 0.05; NS after FDR (*q* = 0.103)
	Work env. ⟶ turnover intention	0.03 (0.02)	[−0.01, 0.07]	0.098 (0.176)	No nominal signal

*Note:* Interaction terms were prespecified and entered simultaneously with main effects in the respective multilevel models. Staffing burden and hierarchy intensity were tested on upstream pathways (candidate proximal variable ⟶ work environment). Organisational readiness (ORIC) was tested on downstream pathways (work environment ⟶ workforce outcomes), as specified in the theoretical framework. *β* = unstandardised interaction coefficient; *p* = uncorrected *p* value; BH *q* = Benjamini–Hochberg false‐discovery‐rate–adjusted q value computed across all nine interaction tests. Four interactions met nominal *p* < 0.05, but none was significant after FDR correction (smallest *q* = 0.054); all results are hypothesis‐generating. The lower confidence‐limit for the readiness × work‐environment term predicting job satisfaction rounds to 0.00; *p* = 0.046 is based on unrounded values. Conditional simple slopes were not reported, so the readiness coefficients are not labelled amplification or attenuation.

Abbreviations: CI = confidence interval, SE = standard error.

### 3.9. Qualitative Findings

Reflexive thematic analysis of focus‐group transcripts (*n* = 36), interview transcripts (*n* = 6), cycle‐reflection records (*n* = 15) and field notes yielded five themes and 15 subthemes. Table 7 presents the thematic structure, excerpts and quantitative integration points. Independent review of a purposively selected 20% subset of transcripts, memos and the evolving thematic map was used to challenge interpretations and refine theme boundaries; no inter‐rater reliability target was applied.

**TABLE 7:** Qualitative thematic framework: themes, subthemes and illustrative evidence.

Theme	Subthemes	Illustrative excerpt	Quantitative integration point
1. From being told to being heard	1a. Shift from directive to dialogic leadership	‘Before, we just followed orders. Now my manager actually asks what we think before changing the schedule’. (FG2, Staff Nurse)	Aligned with increases in psychological safety (*d* = 0.72) and perceived voice (*d* = 0.71)
	1b. Legitimisation of clinical voice	‘I used to keep quiet. After the second cycle, I realised they actually wanted our input’. (FG3, Staff Nurse)	
	1c. Gradual dismantling of silence norms		

2. Trust is built in small moments	2a. Manager follow‐through as a trust signal	‘When she actually changed the handover time as we agreed, that’s when I started to believe this was real’. (FG1, Charge Nurse)	Aligned with increases in relational trust (*d* = 0.59) and manager ability/support (*d* = 0.49)
	2b. Consistency across cycles builds credibility	‘Trust didn’t come from one meeting. It came from seeing the same commitment every cycle’. (INT4, Nurse Manager)	
	2c. Vulnerability reciprocity		

3. Structural walls and workarounds	3a. Staffing constraints limit relational gains	‘We communicate better now, but when you’re covering six patients alone, good communication isn’t enough’. (FG4, Staff Nurse)	Aligned with no staffing indirect‐pathway CI excluding zero; directionally consistent with the nominal pre‐FDR staffing‐burden signal (*β* = −0.04, *p* = 0.012, *q* = 0.054)
	3b. Informal compensatory strategies	‘The staffing issue is beyond our control. We tried to work around it, but honestly, it’s the biggest barrier’. (INT2, Nurse Manager)	
	3c. Frustration with unmodifiable structures		

4. Readiness is not uniform	4a. Prior collaborative culture accelerates change	‘In our unit, we already had some teamwork. The PAR just made it official. But in another unit, they had to start from zero’. (FG2, Staff Nurse)	Contextualises the hierarchy signal (*β* = −0.03, *p* = 0.024, *q* = 0.072) and the two outcome‐specific readiness signals (personal burnout: *p* = 0.008, *q* = 0.054; job satisfaction: *p* = 0.046, *q* = 0.103); none survived FDR correction, and readiness directions require simple‐slope confirmation
	4b. Entrenched hierarchy slows adoption	‘Some managers embraced it from day one. Others needed convincing through two full cycles’. (INT5, Nurse Manager)	
	4c. Administrative support as enabler		

5. Ownership shifts energy	5a. From exhaustion to engagement	‘When you feel like you have a say, the tiredness doesn’t hit the same way. You still work hard, but it feels different’. (FG1, Staff Nurse)	Aligned with longitudinal decreases in burnout (*d* = −0.48) and turnover intention (*d* = −0.46) and an increase in job satisfaction (*d* = 0.49)
	5b. Professional identity reinforcement	‘I was planning to resign. But this process made me feel like I could actually change something here’. (FG3, Staff Nurse)	
	5c. Reduced intention to leave		

*Note:* INT = interview. Excerpts were translated from Arabic and independently reviewed through back‐translation. Quantitative integration references correspond to Tables 3, 4, 5, 6. Five themes and 15 subthemes were developed through combined deductive–inductive reflexive thematic analysis [50]. Independent review challenged interpretations and refined the thematic map; no inter‐rater reliability coefficient was calculated.

Abbreviation: FG = focus group.

Theme 1 (From being told to being heard) captured nurses’ evolving experience from hierarchical, directive leadership toward participatory engagement. Participants described a transition from compliance‐oriented interactions to dialogue‐based exchanges in which clinical observations were solicited and acted upon. This theme corresponded to quantitative improvements in psychological safety and perceived voice. Theme 2 (Trust is built in small moments) described how relational trust between staff nurses and nurse managers accumulated through repeated cycles of responsive action, particularly when managers followed through on commitments from codesign sessions.

Theme 3 (Structural walls and workarounds) captured persistent tension between relational improvements and structural constraints, particularly staffing shortages. Participants described informal strategies to sustain leadership gains under resource pressure while acknowledging that relational improvements could not compensate for inadequate staffing. This aligned with the absence of an indirect‐pathway confidence interval excluding zero for staffing/resource adequacy and was directionally consistent with the nominal pre‐FDR staffing‐burden interaction signal. Theme 4 (Readiness is not uniform) documented how organisational context shaped implementation receptivity: Units with prior collaborative experience adopted changes more readily, while entrenched hierarchy required additional cycles. Theme 5 (Ownership shifts energy) captured the motivational effect of co‐creation, with participants describing greater professional satisfaction and less emotional fatigue when they perceived genuine influence over practice decisions.

### 3.10. Mixed‐Methods Integration

Joint display analysis mapped quantitative trajectories onto qualitative explanatory themes at unit and cross‐site levels. As illustrated in Figure 2, integration generated three categories of meta‐inference: convergence and contextual consistency, complementarity and divergence.

**FIGURE 2:**
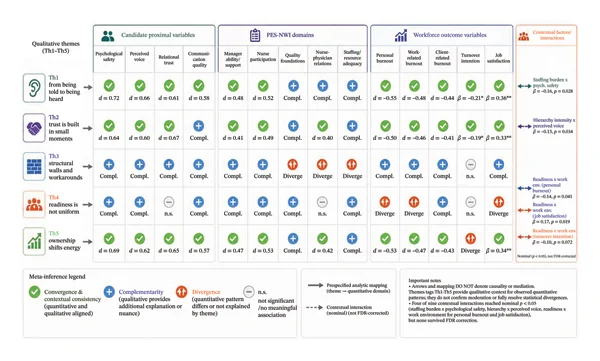
Qualitative–quantitative integration map of verified longitudinal findings, exploratory concurrent change pathways, contextual signals and qualitative themes. Note. this is an integration map rather than a coefficient diagram. Arrows identify prespecified analytic segments and do not represent causal mediation or confirmed moderation. Four of nine contextual interactions met nominal *p* < 0.05: staffing burden × psychological safety, hierarchy intensity × perceived voice, readiness × work environment for personal burnout and readiness × work environment for job satisfaction; none survived Benjamini–Hochberg false‐discovery‐rate correction. Theme tags (Th1–Th5) contextualise but do not statistically validate the quantitative patterns. Verified numerical estimates are reported in Tables 4, 5, 6. The figure was created for this manuscript and contains no third‐party logos or images.

Convergence and contextual consistency. Themes 1, 2 and 5 converged with longitudinal increases in psychological safety, perceived voice and relational trust and with observed workforce trajectories. Themes 3 and 4 contextualised staffing, hierarchy and readiness as potentially relevant conditions. Four interaction coefficients met nominal *p* < 0.05, but none survived FDR correction; qualitative accounts therefore contextualise selected quantitative patterns and do not confirm moderation or resolve the direction of the two readiness interactions.

Complementarity. Qualitative findings elaborated quantitative patterns in three ways. Accounts described early cycles as more disruptive and Cycle 3 as consolidation, contextualising the larger T0–T1 proximal‐variable gains. Theme 3 illuminated why relational improvements did not overcome staffing constraints, consistent with the absence of a staffing/resource change‐pathway interval excluding zero and the missing‐data sensitivity of that domain. Theme 4 described entrenched hierarchy and uneven readiness but did not determine the sign or magnitude of any interaction. Divergence. Readiness patterns were outcome‐specific: The personal‐burnout and job‐satisfaction interactions met nominal *p* < 0.05, whereas readiness × turnover intention did not (*p* = 0.098, *q* = 0.176). Theme 5 included accounts linking voice with intention to remain, but qualitative accounts cannot resolve this statistical divergence. Limited interaction power, individual‐versus‐modelled construct differences, informative departure or chance may contribute; the present data cannot distinguish among them.

### 3.11. Summary of Key Findings

In summary, the PAR‐based programme was associated with favourable change in most measured outcomes, while exploratory client‐related burnout did not change significantly and staffing/resource adequacy was sensitive to missing‐data handling. Psychological safety and perceived voice had the largest number of concurrent change‐pathway intervals excluding zero. Four interactions met nominal pre‐FDR *p* < 0.05, but none survived correction; their directions remain uncertain. Qualitative findings contextualised rather than confirmed these patterns. All findings are associational within an uncontrolled, nonrandomised evaluation.

## 4. Discussion

### 4.1. Principal Findings

This multisite PAR study examined associations between a co‐created supportive leadership programme, nurses’ work environments and workforce outcomes across five Saudi hospitals. Three findings merit cautious emphasis. First, standardised changes were descriptively larger for manager support and nurse participation than for staffing/resource adequacy, although domains were not formally compared and the staffing inference was missing data–sensitive. Second, psychological safety and perceived voice had the largest number of unadjusted concurrent change‐pathway intervals excluding zero; this does not establish mediation. Third, four contextual interactions met nominal *p* < 0.05, including two outcome‐specific readiness terms, but none survived FDR correction and their practical directions remain uncertain.

### 4.2. Differential Domain Responsiveness

The largest estimated standardised changes were observed in nurse manager ability/support and nurse participation, while staffing/resource adequacy showed the smallest estimate and crossed the conventional 0.05 threshold in complete cases. This pattern is theoretically consistent with the architecture of PES‐NWI domains: Managerial support and participatory governance are more directly amenable to local leadership behaviour, whereas staffing adequacy is shaped largely by resource allocation, labour supply and policy constraints beyond unit‐level co‐creation. The pattern is consistent with prior conceptual scholarship but cannot be attributed to the intervention in this uncontrolled evaluation. For evaluation practice, domain‐specific estimates should accompany composite indices so that relational changes are not obscured when structural domains remain comparatively stable. Participant accounts complemented this interpretation by describing perceived changes in trust and communication while characterising staffing constraints as largely beyond local control (Theme 3).

### 4.3. Psychological Safety and Voice as Candidate Proximal Pathway Variables

Psychological safety and perceived voice had the largest number of exploratory concurrent change‐pathway intervals excluding zero. Psychological safety is theoretically relevant to interpersonal risk‐taking and learning [44], and the PAR cycles created structured opportunities for voice through codesign, reflection and anonymous feedback. However, the uncontrolled design, concurrent self‐report changes, unadjusted pathway family and absence of dose‐based exposure preclude mechanistic inference. Larger proximal‐variable gains at T0–T1 than T1–T2 are compatible with early change and later consolidation but do not establish temporal precedence. These patterns are hypotheses for controlled longitudinal studies.

### 4.4. Relational Trust and Communication Quality: Domain‐Specific Candidate Indirect Pathways

Relational trust and process‐level communication quality showed narrower exploratory change‐pathway patterns than psychological safety and voice. Trust products excluded zero for manager support, nurse participation and nurse–physician relations; process‐communication products did so for manager support, nurse participation, foundations for quality and nurse–physician relations. The measurement comparison distinguished process‐level communication quality from the PES‐NWI collegial nurse–physician relations domain in this sample, reducing—but not eliminating—concern about construct overlap. Concurrent, unadjusted products cannot establish mechanism operation.

### 4.5. Selective Contextual Moderation

The interaction results are tentative contextual signals because none survived FDR correction and only five sites were available. The negative staffing‐burden × psychological safety and hierarchy × voice coefficients are compatible with attenuation hypotheses but require conditional slopes and replication. Two readiness × work‐environment coefficients met nominal *p* < 0.05: one for personal burnout and one for job satisfaction. Because higher work‐environment scores are favourable whereas higher burnout scores are unfavourable, the positive burnout coefficient cannot be called amplification without simple slopes; the two readiness patterns are therefore reported as outcome‐specific and directionally unresolved. Theme 4 offers contextual detail but does not validate the coefficients. Future adequately powered studies should test readiness theory [54] with prespecified conditional‐effect analyses.

### 4.6. Workforce Outcomes in the Saudi Nursing Context

Personal and work‐related burnout decreased, whereas exploratory client‐related burnout did not change significantly. Turnover intention decreased and job satisfaction increased among observed/imputed participants, but 19 of 28 endline losses were transfers or resignations, including eight actual resignations. Informative departure and survivor bias therefore remain plausible, and turnover intention findings should not be equated with retention effects. Longer follow‐up using administrative turnover outcomes is needed.

### 4.7. PAR as Implementation Methodology in Hierarchical Clinical Settings

The completion of all planned cycle reviews, 86% endline retention and participant accounts suggest that the PAR process was implementable in these sites. They do not constitute prespecified feasibility or acceptability endpoints. Theme 4 also showed uneven uptake across units. In hierarchical systems, future implementation should test whether longer cycles, explicit readiness assessment and safeguards for equal participation improve the enactment of intended participatory processes.

## 5. Implications for Practice and Policy

For nursing leadership practice, the findings suggest that structured co‐creation can be used to support nurse voice and psychological safety, while its effects require controlled evaluation. The 12‐h facilitator preparation and fidelity checklist are reproducible implementation components, although resource requirements were not formally evaluated. Leadership action should be paired with policy investment in staffing and material resources. Readiness assessment may help tailoring, but its predictive value and direction of effect require confirmation.

## 6. Limitations

Several limitations warrant acknowledgement. First, the uncontrolled single‐arm design precludes causal inference and is vulnerable to secular trends, maturation and reactivity. Second, five hospitals from one regional directorate limit transferability and provide insufficient support for stable hospital random‐slope or between‐site inference. Consistent clinical unit identifiers were unavailable, although many actions and staffing exposures operated at unit level; uncertainty may therefore be underestimated. Third, self‐report and concurrent change measures permit common‐method bias and do not establish temporal mediation. Fourth, 19 of 28 endline losses involved transfer or resignation; the MAR assumption may be violated, and survivor bias may affect workforce conclusions. Fifth, only the nine interactions were FDR‐adjusted; domain estimates, five workforce variables and 20 change‐pathway products were not multiplicity‐adjusted.

Additional limitations concern measurement and qualitative scope. Several adapted Arabic measures were refined and assessed in the same baseline sample used for modelling, and Arabic proficiency was required despite a workforce that was 66% expatriate; selection and cross‐national measurement equivalence were not evaluated. Midpoint timing varied across the March–June window. Four cross‐site focus groups constrained site‐level depth and may have inhibited critical disclosure, and the facilitator–researcher role may have shaped participation and interpretation despite reflexive safeguards. The 12‐month evaluation does not establish durability, measured programme dose was unavailable, and the a priori power calculation addressed within‐person change rather than interaction or site effects. All *p* values and pathway products should therefore be interpreted cautiously.

## 7. Recommendations for Future Research

Future studies should address these limitations by employing cluster‐randomised or stepped‐wedge designs with sufficient clusters to strengthen both causal inference and moderation testing. A 12–24‐month longitudinal follow‐up would assess durability. Multiregion replication across Saudi health directorates or Gulf Cooperation Council states would test transferability. Integrating administrative indicators, actual turnover rates, patient safety events and sick leave patterns with self‐report measures would reduce common‐method variance. Subgroup analyses by nationality, clinical speciality and role stratum would clarify whether intervention effects vary across the diverse segments of the Saudi nursing workforce. Finally, a cost‐effectiveness analysis of the PAR‐based model relative to conventional leadership training programs would inform resource‐allocation decisions for healthcare organisations.

## 8. Conclusion

This multisite PAR evaluation found favourable longitudinal associations in most work‐environment and workforce measures across five Saudi hospitals. Psychological safety and perceived voice had the largest number of exploratory concurrent change‐pathway intervals excluding zero, but they are not confirmed mediators. Standardised changes were descriptively larger for relational and participatory domains than for staffing/resource adequacy, whose borderline inference was sensitive to missing‐data handling. Four contextual interactions met nominal pre‐FDR *p* < 0.05—including two outcome‐specific readiness terms—but none survived correction, and their directions remain unresolved.

These findings justify further controlled testing of participatory leadership models that enable nurse voice and interpersonal safety while pairing local action with staffing and resource policy. Readiness may inform tailoring, but predictive benefit has not been established. Because the evaluation was uncontrolled, nonrandomised, subject to informative attrition, and based on only five sites, conclusions remain associational. Cluster‐randomised or stepped‐wedge studies with more clusters, objective workforce indicators, explicit dose measurement and longer follow‐up are required.

## Author Contributions

Majed Mowanes Alruwaili conceived and designed the study, developed the theoretical framework, obtained ethical approvals, coordinated data collection, performed quantitative and qualitative analyses, interpreted the findings, and drafted and critically revised the manuscript.

## Funding

This work was funded by the Deanship of Graduate Studies and Scientific Research at Jouf University under Grant No. DGSSR‐2025‐01‐01585.

## Ethics Statement

Ethical approval was obtained from the Jouf University Institutional Review Board (Protocol No. 7603) and from the ethics committees of all five participating hospitals prior to recruitment. All procedures were performed in accordance with the ethical standards of the institutional and national research committees and with the 1964 Declaration of Helsinki and its later amendments.

## Consent

Written informed consent was obtained from all participants before the commencement of data collection. Participants were informed of their right to withdraw at any stage without occupational consequences.

## Conflicts of Interest

The author declares no conflicts of interest.

## Supporting Information

Additional supporting information can be found online in the Supporting Information section.

## Supporting information


**Supporting Information** Supporting Tables S1–S4 provide additional methodological and sensitivity‐analysis information. Supporting Table S1 summarises instrument sources, scoring, adaptation procedures, baseline reliability, and dimensionality and validity evidence. Supporting Table S2 describes the qualitative data‐collection tools, their development and pilot refinement, and quality‐assurance procedures. Supporting Table S3 reports baseline interconstruct correlations and the discriminant‐validity comparison between process‐level communication quality and the PES‐NWI collegial nurse–physician relations domain. Supporting Table S4 presents the complete‐case sensitivity analysis for the three‐level mixed‐effects models.

## Data Availability

De‐identified quantitative data and analysis syntax may be available from the corresponding author following institutional review, a methodologically sound proposal and an approved data‐use agreement. Qualitative transcripts are not publicly shared because contextual detail could permit participant or site identification.
