# Supplementary material for: Co‐Creating Supportive Leadership and Nurse Work Environments: A Mixed‐Methods Participatory Action Research Study in Saudi Arabia
Source: J Nurs Manag. 2026 Sep 27;2026:7257178. doi: 10.1155/jonm/7257178 (PMC13617313; doi:10.1155/jonm/7257178)
Supplement: Supplementary file 1 [file JONM-2026-7257178-s001.pdf]

Supplementary Table S1. Study Instruments: Source, Adaptation, Scoring, and Psychometric Properties

Instruments are organised by theoretical-framework role (work-environment outcomes, workforce outcomes, candidate proximal variables, and contextual variables). Measures marked 'Full 7-step protocol' underwent the standardised translation, adaptation, and preliminary within-sample psychometric workflow described in the Methods. Baseline psychometric values are from the enrolled sample (n = 200) unless otherwise specified.

Construct	Instrument	Developer(s)	Items	Scale Type	Score Range & Direction	Subscales	Adaptation Procedures	Baseline α	Dimensionality & Validity Evidence	Retained
Work-Environment Outcomes (primary composite; exploratory domains)										
Practice environment	*PES-NWI*	Lake, 2002	31	4-point Likert (1–4)	1.00–4.00; higher = more favorable environment	Nurse Manager Ability (5)<br>Nurse Participation (9)<br>Foundations for Quality (10)<br>Nurse–Physician Relations (3)<br>Staffing & Resources (4)	Arabic version (Ambani et al., 2019) used.<br>Item wording harmonized for Jouf regional dialect; no structural changes.	α total = .91<br>Subscales: .84, .86, .88, .79, .81	Prior CFA in Arabic samples confirmed 5-factor structure.<br>Item–total correlations .42–.71.<br>Inter-subscale r = .31–.62.	Yes
Workforce Outcome Variables (four secondary endpoints; client-related burnout exploratory)										
Burnout	*CBI*	Kristensen et al., 2005	19	5-point frequency (0–100)	0–100 per subscale; higher = greater burnout	Personal burnout (6)<br>Work-related burnout (7)<br>Client-related burnout (6)	Arabic-language form used; reliability verified in the present sample.	α subscales: .87, .84, .82	Three-factor structure confirmed in Arabic nursing populations.<br>Convergent validity supported by correlation with emotional exhaustion (r = .74).	Yes
Turnover intention	*TIS-6*	Bothma & Roodt, 2013	6	5-point Likert (1–5)	6–30; higher = stronger turnover intention	None (unidimensional)	Arabic form applied in Saudi nursing contexts.<br>Minimal language adaptation; comprehension verified in pilot (n = 20).	α = .83	Unidimensional structure confirmed (single factor; loadings .58–.79).<br>Intention to stay derived by reverse scoring.	Yes
Job satisfaction	Adapted MSQ-SF	Weiss et al., 1967	14	5-point Likert (1–5)	14–70; higher = greater satisfaction	Intrinsic satisfaction (8)<br>Extrinsic satisfaction (6)	Full 7-step protocol.<br>20 items initially translated; 6 items removed after expert panel (3) and pilot (3) due to low clarity or cultural mismatch.<br>Pilot (n = 20): comprehension satisfactory after 4 wording refinements.	α total = .88<br>Intrinsic: .84<br>Extrinsic: .81	EFA supported 2-factor solution (67.4% variance explained).<br>All retained item loadings ≥ .52.<br>Item–total correlations .44–.69.	Yes

Construct	Instrument	Developer(s)	Items	Scale Type	Score Range & Direction	Subscales	Adaptation Procedures	Baseline α	Dimensionality & Validity Evidence	Retained
Candidate Proximal Variables										
Psychological safety	Adapted TPS	Edmondson, 1999	6	7-point Likert (1–7)	6–42; higher = greater psychological safety	None (unidimensional)	Full 7-step protocol.<br>7 items initially translated; 1 item removed after pilot (ambiguous wording in Arabic).<br>Items reframed from 'team' to 'nursing unit' context.<br>Pilot (n = 20): no comprehension issues after refinement.	α = .79	Single-factor EFA solution (52.8% variance explained).<br>Loadings .48–.76.<br>Item–total correlations .39–.61.<br>Discriminant r with relational trust = .54 (below .85 threshold).	Yes
Perceived voice	Adapted Voice Scale	LePine & Van Dyne, 1998	5	7-point Likert (1–7)	5–35; higher = greater perceived openness to voice	None (unidimensional)	Full 7-step protocol.<br>Original 6 behavioral items reframed to employee-perception format ('In my unit, it is safe to...').<br>1 item removed post-expert review (redundancy with psychological safety item).<br>Pilot (n = 20): satisfactory clarity.	α = .82	Single-factor solution (58.3% variance).<br>Loadings .56–.81.<br>Discriminant r with psychological safety = .61 (below .85).	Yes
Relational trust	Adapted Trust Scale	McAllister, 1995	8	7-point Likert (1–7)	8–56; higher = greater trust in nurse manager	Affect-based trust (4)<br>Cognition-based trust (4)	Full 7-step protocol.<br>11 items initially selected; 3 removed (2 by expert panel for cultural mismatch; 1 by pilot for ambiguity).<br>Items focused on staff nurse–nurse manager dyad.<br>Pilot (n = 20): 2 wording refinements made.	α total = .86<br>Affect: .83<br>Cognition: .80	Two-factor EFA consistent with original (69.1% variance).<br>Loadings .54–.82.<br>Inter-factor r = .58.<br>Discriminant r with psychological safety = .54.	Yes
Process-level communication quality	Adapted Comm-Q	Shortell et al., 1991<br>+ healthcare team items	7	5-point Likert (1–5)	7–35; higher = better interpersonal communication quality	None (unidimensional)	Full 7-step protocol.<br>9 items initially drawn from Shortell subscale (5) and healthcare teamwork literature (4).<br>2 items removed post-pilot (low relevance to direct nurse–nurse/nurse–manager exchanges).<br>Pilot (n = 20): comprehension satisfactory.	α = .81	Single-factor solution (54.6% variance).<br>Loadings .51–.78.<br>Discriminant validity versus the PES-NWI collegial nurse–physician relations domain: r = .47; CFA $\Delta\chi^2$ supported the two-construct over the single-construct model ($p < .001$).	Yes

Construct	Instrument	Developer(s)	Items	Scale Type	Score Range & Direction	Subscales	Adaptation Procedures	Baseline α	Dimensionality & Validity Evidence	Retained
Contextual Variables										
Organizational readiness	*ORIC*	Shea et al., 2014	12	5-point Likert (1–5)	12–60; higher = greater readiness	Change commitment (5)<br>Change efficacy (7)	7-step adaptation protocol applied.<br>All 12 items retained after expert review and pilot.<br>Pilot (n = 20): no comprehension issues.	α total = .89<br>Commitment: .85<br>Efficacy: .86	Two-factor structure supported (71.3% variance).<br>Loadings .59–.84.<br>Inter-factor r = .63.	Yes
Staffing burden	Nurse-to-patient ratio	Administrative data	N/A	Continuous ratio	Higher ratio = greater patient burden per nurse	N/A	Extracted per unit per wave from hospital HR/scheduling records.<br>Standardized extraction protocol across 5 sites.	N/A	Face validity via operational definition; cross-checked against CBAHI staffing records.	Yes
Hierarchy intensity	Adapted Hierarchy Scale	Dorfman & Howell, 1988	5	5-point Likert (1–5)	5–25; higher = stronger perceived hierarchy	None (unidimensional)	Full 7-step protocol.<br>8 items initially selected from power-distance/workplace authority literature.<br>3 removed: 2 by expert panel (low relevance to nurse–manager dynamics); 1 by pilot (double-barreled).<br>Pilot (n = 20): 1 wording refinement.	α = .77	Single-factor solution (49.2% variance).<br>Loadings .44–.73.<br>Item–total correlations .36–.58.	Yes

Abbreviations: PES-NWI, Practice Environment Scale of the Nursing Work Index; CBI, Copenhagen Burnout Inventory; TIS-6, Turnover Intention Scale–6; MSQ-SF, Minnesota Satisfaction Questionnaire—Short Form; TPS, Team Psychological Safety; Comm-Q, Communication Quality; ORIC, Organizational Readiness for Implementing Change; EFA, exploratory factor analysis; CFA, confirmatory factor analysis; N/A, not applicable; CBAHI, Saudi Central Board for Accreditation of Healthcare Institutions.

Notes: (1) Measures marked 'Full 7-step protocol' followed the standardised translation, adaptation, and preliminary within-sample psychometric workflow detailed in the Methods. (2) The pilot sample (n = 20) was drawn from eligible nurses at study hospitals but excluded from the analytic cohort. (3) Baseline psychometric values are from the enrolled sample (n = 200). (4) Prespecified adequacy thresholds were α ≥ .70, primary factor loadings ≥ .40, and inter-construct r < .85. (5) Parenthetical numbers after subscale names indicate item counts. (6) Measurement distinction between process-level communication quality (candidate proximal variable) and the PES-NWI collegial nurse–physician relations domain was examined before exploratory change-pathway modelling.

Supplementary Table S2. Qualitative Data Collection Tools: Design, Content, Development, and Quality Assurance

Tool	Target Participants	Sessions / Use	Content Domains (Framework-Aligned)	Development & Review Procedure	Pilot / Refinement	Quality Assurance Measures
Frontline-nurse focus-group guide	Staff and charge nurses (n = 36; 4 groups of 8–10)	4 sessions (T1 and T2 cycle points)	1. Baseline work-environment experiences and barriers (Prop. 1–2)<br>2. Perceived changes in leadership behaviour and support (Prop. 1)<br>3. Psychological safety, voice, and trust experiences (Prop. 2)<br>4. Communication quality within unit teams (Prop. 2–3)<br>5. Staffing and resource constraints (contextual factors)<br>6. Perceived changes in satisfaction, burnout, and intention to stay (Prop. 3–4)	Developed deductively from framework propositions.<br>Reviewed by 3 expert qualitative nursing researchers (content relevance, clarity, cultural sensitivity).<br>Revised based on expert consensus.	Piloted with 2 staff nurses (non-cohort).<br>Refinements: 1 question reworded for clarity; 1 probe added for cultural context.	Version-controlled (3 versions documented).<br>All iterations in audit trail.<br>Guides used consistently across sites with facilitator standardization notes.
Nurse-manager semi-structured interview guide	Nurse managers (n = 6; individual interviews)	6 interviews (T2 endline)	1. Co-design rationale and decision-making processes (Prop. 1)<br>2. Implementation challenges and facilitators (process)<br>3. Observed changes in unit dynamics and staff behaviour (Prop. 2–3)<br>4. Leadership-role evolution during PAR cycles (Prop. 1)<br>5. Hierarchy and authority dynamics (contextual factor)<br>6. Sustainability perceptions and organisational readiness (Prop. 4)	Developed deductively from framework.<br>Expert-reviewed by same 3-member panel.<br>Cross-referenced with focus-group guide for complementarity.	Piloted with 1 nurse manager (non-cohort).<br>Refinement: 1 question split into two for specificity; probes added for hierarchy domain.	Version-controlled (2 versions).<br>Documented in audit trail.<br>Interview duration: 40–60 minutes.<br>Audio-recorded, transcribed verbatim, accuracy-checked.
Standardized cycle reflection template	PAR teams (nurses + nurse managers per site)	15 site-cycle reflections (3 cycles × 5 hospitals)	1. Actions implemented this cycle (description, owner, timeline)<br>2. Feasibility assessment (ease of integration into workflow)<br>3. Uptake indicators (participation rates, adoption signals)<br>4. Barriers encountered (structural, relational, resource-based)<br>5. Perceived effects on target proximal mechanism<br>6. Unintended consequences (positive and negative)<br>7. Recommended adaptations for next cycle	Study-developed based on PAR process logic.<br>Expert-reviewed for completeness and usability.<br>Iteratively refined after Cycle 1 feedback (added 'unintended consequences' section).	Not independently piloted (template refined iteratively within implementation).<br>Post-Cycle 1 revision: added structured prompts for mechanism linkage.	Standardized across all 5 sites.<br>Completed by PAR team lead after each cycle debrief.<br>Archived chronologically for cross-cycle comparison.<br>Supports dependability and confirmability.
Field-note and cycle-log protocol	Research team / PAR facilitators	Continuous use across 12-month implementation	1. Process observations (session dynamics, participation patterns, power dynamics)<br>2. Contextual events (staffing changes, policy shifts, external disruptions)<br>3. Implementation fidelity observations (adherence to cycle phases)<br>4. Emerging analytic notes and hypotheses<br>5. Reflexive observations (facilitator positionality, assumptions)	Study-developed standardized template.<br>Structured sections aligned to PAR fidelity checklist domains.<br>Reflexive section guided by Bradbury (2015) PAR quality criteria.	Template standardized before implementation.<br>No pilot needed (researcher-completed tool); format adjusted after first month for usability.	Continuous chronological record across all sites.<br>Reviewed during post-cycle debriefs.<br>Archived as core audit trail document.<br>Supports confirmability and decision transparency.

Abbreviations: PAR, Participatory Action Research; Prop., Proposition (from the Theoretical Framework).

Notes: (1) All guides were developed in Arabic. (2) Proposition numbers refer to the four directional propositions specified in the Theoretical Framework. (3) Version control included date-stamped iterations with change logs maintained in the study audit trail. (4) All qualitative sessions were conducted in Arabic by trained facilitators. (5) Transcription accuracy was verified by a second team member against the original audio recording.

Supplementary Table S3. Inter-Construct Correlation Matrix for Candidate Proximal Variables, the PES-NWI Comparator, and Job Satisfaction at Baseline (n = 200)

Construct	1	2	3	4	5	6
1. Psychological safety	—					
2. Perceived voice	.61**	—				
3. Relational trust	.54**	.52**	—			
4. Process communication quality (candidate proximal variable)	.49**	.46**	.58**	—		
5. Collegial nurse–physician relations (PES-NWI domain)	.41**	.38**	.44**	.47**	—	
6. Job satisfaction	.53**	.48**	.57**	.51**	.55**	—

** $p < .01$ (two-tailed).

Notes: All correlations were below the prespecified $r < .85$ discriminant-validity threshold. The critical comparison—process-level communication quality (Construct 4, candidate proximal variable) versus collegial nurse–physician relations (Construct 5, PES-NWI outcome)—yielded $r = .47$. A two-factor model fit better than a one-factor model, $\Delta\chi^2(1) = 34.2$, $p < .001$ (two-factor CFI = .94, RMSEA = .06; one-factor CFI = .81, RMSEA = .11), supporting measurement distinction in this sample without establishing causal pathway separation.

Supplementary Table S4. Sensitivity Analysis: Complete-Case Three-Level Mixed-Effects Model Estimates (*n* = 172 Completers)

This table replicates the longitudinal analyses reported in Table 4 using only the 172 participants who completed all three measurement waves, without multiple imputation. Model specifications are identical to Table 4: three-level mixed-effects models with participant-level random intercepts (Level 2) and hospital-level random intercepts (Level 3). Hospital-level random slopes for time were tested and retained where model fit improved. The purpose is to assess sensitivity of the reported estimates to the multiple-imputation strategy.

Outcome	T0 → T1			T0 → T2			Cohen's *d*	Hospital ICC
	β (SE)	95% CI	*p*	β (SE)	95% CI	*p*		
Work-Environment Outcomes (PES-NWI)								
**Composite score**	0.07 (0.03)	[0.01, 0.14]	.012	0.17 (0.04)	[0.09, 0.25]	<.001	0.35	.03
Manager ability & support	0.11 (0.04)	[0.03, 0.20]	.004	0.26 (0.05)	[0.16, 0.36]	<.001	0.47	.04
Nurse participation	0.10 (0.04)	[0.02, 0.18]	.008	0.22 (0.05)	[0.12, 0.32]	<.001	0.41	.03
Foundations for quality	0.07 (0.03)	[0.01, 0.14]	.018	0.15 (0.04)	[0.07, 0.23]	<.001	0.33	.02
Nurse–physician relations	0.06 (0.04)	[−0.02, 0.14]	.098	0.14 (0.05)	[0.04, 0.24]	.006	0.27	.03
Staffing & resource adequacy	0.05 (0.04)	[−0.03, 0.13]	.186	0.12 (0.06)	[0.00, 0.24]	.054	0.21	.05
Workforce Outcomes								
Personal burnout (CBI)	−5.1 (1.5)	[−8.0, −2.2]	<.001	−7.2 (1.7)	[−10.5, −3.9]	<.001	−0.46	.03
Work-related burnout	−4.0 (1.5)	[−7.0, −1.0]	.008	−6.3 (1.8)	[−9.8, −2.8]	<.001	−0.38	.04
Client-related burnout	−2.1 (1.7)	[−5.4, 1.2]	.204	−3.5 (2.1)	[−7.6, 0.6]	.092	−0.19	.02
Turnover intention (TIS-6)	−1.0 (0.4)	[−1.8, −0.2]	.014	−2.0 (0.5)	[−2.9, −1.1]	<.001	−0.44	.04
Job satisfaction	2.3 (0.8)	[0.7, 3.9]	.004	4.1 (1.0)	[2.2, 6.0]	<.001	0.47	.03

Note. Complete-case analysis was restricted to participants who completed all three measurement waves (*n* = 172). β = unstandardised fixed-effect estimate for time contrast (reference: T0); SE = standard error; CI = confidence interval; *d* = standardised T0–T2 mean change, computed as the model-estimated change divided by the pooled T0 and T2 observed standard deviation; ICC = intraclass correlation at hospital level. Model specification was identical to the primary imputed analysis (Table 4): participant-level and hospital-level random intercepts; hospital-level random slopes for time were retained where fit improved by likelihood-ratio testing. Confidence-limit rounding may display 0.00 for a value close to zero.

Concordance Summary

Of 11 T0–T2 outcome tests, 10 retained the same conventional $p < .05$ classification as the primary imputed analysis (Table 4). One outcome showed minor attenuation consistent with the reduced sample size:

Staffing and resource adequacy shifted from $p = .038$ in the imputed analysis (Table 4) to $p = .054$ in the complete-case analysis. This outcome had the smallest standardised change among the work-environment outcomes ($d = 0.21$–0.23); the direction and magnitude were similar, but the confidence interval included zero in the complete-case analysis. The inference for this borderline domain is therefore sensitive to the missing-data approach.

For nurse–physician relations, the T0–T1 estimate remained non-significant in both analyses ($p = .072$ imputed; $p = .098$ complete case), while the T0–T2 estimate remained significant ($p = .004$ imputed; $p = .006$ complete case). This did not represent a change in the T0–T2 classification.

The PES-NWI composite, manager ability/support, nurse participation, foundations for quality, nurse–physician relations, personal burnout, work-related burnout, turnover intention, and job satisfaction retained T0–T2 confidence intervals excluding zero with comparable standardised changes (absolute $\Delta d \leq 0.02$). Client-related burnout retained a confidence interval including zero. The complete-case analysis therefore supports the robustness of most reported estimates while showing that the staffing/resource-adequacy inference is sensitive to the missing-data approach.
